# Aberrant DNA Methylation of P16, MGMT, and hMLH1 Genes in Combination with MTHFR C677T Genetic Polymorphism in gastric cancer

**DOI:** 10.12669/pjms.296.3711

**Published:** 2013

**Authors:** Binbin Song, Jiang Ai, Xianghong Kong, Dexin Liu, Jun Li

**Affiliations:** 1 Binbin Song, Department of Gastroenterology, Hongqi Hospital, Mudanjiang, China.; 2 Jiang Ai, Department of Gastroenterology, Hongqi Hospital, Mudanjiang, China.; 3 Xianghong Kong, Department of Gastroenterology, Hongqi Hospital, Mudanjiang, China.; 4 Dexin Liu, Department of Gastroenterology, Hongqi Hospital, Mudanjiang, China.; 5 Jun Li, Affiliated Hospital of Sun Yat-sen University of Huzhou, Huizhou, China.

**Keywords:** Aberrant DNA Methylation, P16, MGMT, hMLH1, Gastric cancer

## Abstract

***Objective:*** We aimed to explore the association of P16, MGMT and HMLH1 with gastric cancer and their relation with Methylenetetrahydrofolate reductase (MTHFR).

***Methods:*** 322 gastric patients who were confirmed with pathological diagnosis were included in our study. Aberrant DNA methylation of P16, MGMT and HMLH1 and polymorphisms of MTHFR C677T and A1298C were detected using PCR-RFLP.

***Results:*** The proportions of DNA hypermethylation in P16, MGMT and hMLH1 genes in gastric cancer tissues were 75.2% (242/322), 27.6% (89/322) and 5.3% (17/322), respectively. In the remote normal-appearing tissues, 29.5% (95/322) and 16.1%(52/322) showed hypermethylation in P16 and MGMT genes, respectively. We found a significantly higher proportion of DNA hypermethylation of P16 in patients with N1 TNM stage in cancer tissues and remote normal-appearing tissues (*P*<0.05). Similarly, we found DNA hypermethylation of MGMT had significantly higher proportion in N1 and M1 TNM stage (*P*<0.05). Individuals with homozygotes (TT) of MTHFR C677T had significant risk of DNA hypermethylation of MGMT in cancer tissues [OR (95% CI)=4.27(1.76-7.84)], and a significant risk was also found in those carrying MTHFR 677CT/TT genotype [OR (95% CI)= 3.27(1.21-4.77)].

***Conclusion:*** We found the aberrant hypermethylation of cancer-related genes, such as P16, MGMT and HMLH1, could be predictive biomarkers for detection of gastric cancer.

## INTRODUCTION

Gastric cancer is one of the most common cancers worldwide, and it ranks the second leading cause of cancer-related deaths after lung cancer.^1^ Almost two-thirds of gastric cancer cases and deaths occur in less developed regions.^1^ In China, there were almost 0.4 million new cases from gastric cancer, and rank the third most common cancer.^1^ Infection with H.pylori is a well-established cause of gastric cancer, but variants in various genetic factors also influence the susceptibility of gastric cancer.^2^

It is reported that dietary factors, either deficiencies or excesses of nutrients during the process of one-carbon metabolism pathway, have been associated with increased risk of gastric cancer.^3^ The one-carbon cycle plays an important role in cellular proliferation and epigenetic modification, and folate is a important component of this pathway.^3^ Folate functions as a critical source of carbon moieties in the synthesis of nucleotides, DNA repair and replication. These effects of foalte may affect the susceptibility of gastric cancer.^4^^,^^5^ Methylenetetrahydrofolate reductase (MTHFR) is a critical enzyme in folate metabolism, is responsible for the circulation form of folate and 5-methyltetrahydrofolate which is involved in DNA synthesis and methylation.^6^ The activity of MTHFR is controlled mainly by the genetic polymorphisms and shows variation between different individuals.^7^^,^^8^ MTHFR C677T (rs1801133) and MTHFR A1298C (rs1801131) polymorphisms are associated with a reduced activity of MTHFR.

DNA methylation is an important epigenetic feature of DNA, which has an important role in eukaryotes, gene regulation, cellular differentiation mechanisms, X chromosome inactivation, aging and carcinogenesis.^9^ Alteration of DNA methylation in genome can be found in various cancers, and induce the over-expression of oncogenes and silencing of tumor suppressor genes in the process of carcinogenesis. P16, MGMT and HMLH1 are important tumor suppressor and DNA repair genes, and have involved in the carcinogenic process. Inactivation of P16, MGMT and HMLH1 plays a role in the progression of various cancers, and the inactivation of them is induced by aberrant hypermethylation. However, the related information for gastric cancer are still limited.^10^ Therefore, we aimed to explore the association of P16, MGMT and HMLH1 with gastric cancer and their relation with MTHFR.

## METHODS

This study recruited gastric cancer patients who were confirmed with pathological diagnosis in Huizhou Municipal Central Hospital of Guangdong between March 2009 and December 2011. Patients who had cardiac adenocarcinoma, secondary or recurrent tumors, a history of other malignant neoplasms, and previous eradication therapy for H.pylori were excluded. Our study was approved by Ethnic committee of Hongqi Hospital and all the patients signed the consent form.

All the patients underwent surgery, cancer lesion and remote normal-appearing tissues were excised and stored at -70ºC in Liquid Nitrogen refrigerator immediately until use. Twenty normal gastric tissue samples were obtained under surgery and also stored. H.pylori genotyping.

All the patients were required to provide 5ml peripheral bloods before surgery, and the bloods were stored at -20ºC. Enzyme linked immunoabsorbent assay (ELISA) was used for qualitative determination of IgG antibodies (HpIgG ELISA) to Helicobacter pylori in serum. The process of experiment and determination of results were according to manufacturer’s instructions of the commercially available kit (Genesis Diagnostics, Cambridgeshire, UK). 

***DNA extraction and quantification: ***5 ml venous blood was drawn from each cases and controls. The blood was kept in -20 ºC, and EDTA with 1.5~2.2mg/ml was used for anticoagulant. Total DNA was extracted from the buffy-coat layer using a TIANamp blood DNA kit (Tiangen Biotech, Beijing, China) with centrifuging for 3 minutes at 13.400 x g (12.000 rpm). The methylation of P16, MGMT and hMLH1 was determined by the method of methylation-specific PCR after sodium bisulfate modification of DNA (Wang et al., 2008; Herman et al., 1996^1^). The pairs of primers were designed using Assay Design 3.1 software (Sequenom, San Diego, CA, USA; Table-I).^11^ The 1.5 to 2.0 ug of genomic DNA was dissolved in H_2_O and incubated into 5.5uL NaOH for 10 minutes at 37°C, and then treated by hydroquinone and NaHSO_3_.After these procedures, the unmethylated cytosine would be converted to uracil and determined as thymine by Taq polymerase during the PCR process according to the instruction.

Genotyping of MTHFR C677T and MTHFR A1298C polymorphism was determined using Polymerase Chain Reaction-Restriction Fragment Length Polymorphism (PCR-RFLP) according to previous report.^12^ Each PCR reaction mix comprised 50 ng genomic DNA, 200 μM dNTP, 2.5 U Taq DNA polymerase (Promega, Madison, WI, USA), and 200 μM primers, in a total volume of 20 µl. The cycling programme involved preliminary denaturation at 94°C for two minutes, followed by 35 cycles of denaturation at 94°C for 30 s, and annealing at 64°C for 30 s, with a final extension at 72°C for 10 minutes. PCR products were verified by 1.0% agarose gel electrophoresis, and the PCR products were visualized using ethidium bromide staining.

**Table-I:** Primers in the PCR process

*Gene*	*Primer*		*Sequence(5’→3’)*
P16	M	F	TTATTAGAGGGTGGGGCGGATCGC
		B	GACCCCGAACCGCGACCGTAA
	U	F	TTATTCGCGGGTGGGGTGGATTGT
		B	CAACCCCAAACCACAACCATAA
MGMT	M	F	TTTCGATTCGTAGGTTCGCCGC
		B	GCACTCTTCCGAAAACGAAACG
	U	F	GTGTTTTGATGTTTGTAGGTTTTTGT
		B	TCCACACTCTTCCAAAAACAAAACA
hMLH1	M	F	ACGTAGACGTTTTATTAGCGC
		B	CCTCATCGTAACTACCCGCGC
	U	F	TTTTGATGTAGATGTTTTAGG
		B	ACCACCTCATCATAACATCCC
MTHFR C677T		F	CGTGGCTCCTGCGTTTCC
		B	GAGCCGGCCACAGGCAT
MTHFR A1298C		F	AAATGATCTGGGAGCTGAGT
		B	CAGAGTATATGGCAGTACAG

M: Methylated; U: Unmethylated; F: Forward; B: Backward.

***Statistical analysis: ***All statistical analyses were performed using SPSS® version 11.0 (SPSS Inc., Chicago, IL, USA) for Windows®. Continuous variables were presented as mean±SD and analysed using independent sample t-test. Categorical variables were presented as n of subjects (%) and analysed using χ2-test. Odds ratios (OR) and their corresponding 95% confidence intervals (CI) were used to assess the influence of MTHFR on the. All comparisons were two-sided, and *P *< 0.05 was regarded as statistically significant.

## RESULTS

A total of 348 gastric cancer patients were included in our study, and 322 patients were involved in the final analysis (participation rate: 92.5%; 173 males and 149 females). The average age of 322 patients were 54.5±8.5 years old. The DNA hypermethylation of P16, MGMT and hMLH1 in cancer tissue and paracancerous normal tissue was shown in Table-II. The proportions of DNA hypermethylation in P16, MGMT and hMLH1 genes in gastric cancer tissues were 75.2% (242/322), 27.6% (89/322) and 5.3% (17/322), respectively. In the remote normal-appearing tissues, 29.5% (95/322) and 16.1%(52/322) showed hypermethylation in P16 and MGMT genes, respectively. The proportion of DNA hypermethylation in P16, MGMT and hMLH1 in cancer tissues were significantly higher than remote normal-appearing tissues. We did not find significant association of DNA hypermethylation with sex, and tumor sites either in gastric cancer tissues or remote normal-appearing tissues. 

The hypermethylation of P16 and MGMT showed significant correlation with the different clinical characteristics (Table-II). We found a significantly higher proportion of DNA hypermethylation of P16 in patients with N1 TNM stage in cancer tissues and remote normal-appearing tissues (*P*<0.05). Similarly, we found DNA hypermethylation of MGMT had significantly higher proportion in N1 and M1 TNM stage (*P*<0.05).

The association of DNA hypermethylation with MTHFR C677T and C1298A polymorphisms is shown in Table-III. Our finding showed that individuals with homozygotes (TT) of MTHFR C677T had significant risk of DNA hypermethylation of MGMT in cancer tissues [OR (95% CI)=4.27(1.76-7.84)], and a significant risk was also found in those carrying MTHFR 677CT/TT genotype [OR (95% CI)= 3.27(1.21-4.77)]. However, we did not find association between polymorphisms in MTHFR A1298C and risk of DNA hypermethylation of P16, MGMT and hMLH1 gene.

**Table-II:** Association of DNA hypermethylation with demographic and clinical characteristics of gastric cancer

*Variables*	*N=322*	*Cancer tissue*						*Remote normal-appearing tissues*					
		*P16*	*%*	*MGMT*	*%*	*hMLH1*	*%*	*P16*	*%*	*MGMT*	*%*	*hMLH1*	*%*
*Mean age (years)*	54.5±8.5												
Sex													
Male	173	125	72.3	49	28.3	9	5.2	50	28.9	28	16.2	0	0
Female	149	117	78.5	40	26.8	8	4.6	45	26.0	24	16.1	0	0
*P* value		0.91		0.77		0.95		0.81		0.99		1.0	
**Site**													
Upper	60	44	73.5	17	28.4	3	1.7	17	9.8	10	16.7	0	0
Middle	153	114	74.5	41	26.8	9	5.2	44	25.4	24	15.7	0	0
Low	109	84	77.0	31	28.4	5	2.9	34	19.7	18	16.5	0	0
*P* value		0.84		0.95		0.89		0.89		0.98		1.0	
*TNM stage*													
*T*													
T1	44	30	68.5	10	22.8	2	1.2	12	6.9	6	13.7	0	0
T2	95	69	72.4	25	26.3	4	2.3	24	13.9	12	12.6	0	0
T3	99	74	74.9	25	25.3	5	2.9	26	15.0	13	13.2	0	0
T4	84	69	82.4	29	34.4	6	3.5	33	19.1	21	24.9	0	0
*P* value		0.3		0.41		0.84		0.15		0.09		1.0	
*N*													
N0	180	125	69.4	41	22.8	8	4.6	51	29.5	28	15.6	0	0
N1	142	117	82.4	48	33.8	9	5.2	44	25.4	24	16.9	0	0
*P* value		0.008*		0.007*		0.45		0.001*		0.03*		1.0	
*M*													
M0	294	221	75.2	74	25.2	15	8.7	84	48.6	45	15.3	0	0
M1	28	21	75.0	15	53.5	2	1.2	11	6.4	7	25.0	0	0
*P* value		0.98		0.001*		0.64		0.24		0.18		1.0	

**Table-III:** Association of DNA hypermethylation with MTHFR polymorphisms

*Variables*	*OR (95% CI)* ^1^					
	*Cancer tissue*			*Remote normal-appearing tissues*		
	*P16*	*MGMT*	*hMLH1*	*P16*	*MGMT*	*hMLH1*
*MTHFR C677T*						
CC	1.0(Ref.)	1.0(Ref.)	1.0(Ref.)	1.0(Ref.)	1.0(Ref.)	-
CT	1.03(0.65-2.86)	1.41(0.75-2.89)	1.20(0.30-9.7)	0.98(0.34-7.87)	1.63(0.34-9.14)	-
TT	1.68(0.87-5.54)	4.27(1.76-7.84)	1.72(0.45-15.7)	1.55(0.57-7.14)	2.43(0.89-10.46)	-
CT/TT	1.450.82-2.32)	3.27(1.21-4.77)	1.45(0.34-13.1)	1.32(0.57-6.98)	2.10(0.77-7.31)	-
*MTHFR A1298C*						
AA	1.0(Ref.)	1.0(Ref.)	1.0(Ref.)	1.0(Ref.)	1.0(Ref.)	-
AC	1.12(0.53-3.32)	1.23(0.64-2.34)	1.05(0.21-11.4)	1.04(0.36-4.70)	1.35(0.35-4.54)	-
CC	1.32(0.68-3.73)	2.41(0.93-4.72)	1.30(0.32-12.7)	1.41(0.44-6.78)	2.17(0.45-9.33)	-
AC/CC	1.25(0.70-3.15)	1.84(0.85-3.79)	1.18(0.30-12.1)	1.21(0.53-5.58)	1.76(0.51-6.40)	-

## DISCUSSION

Varied activity of folate metabolic enzyme which induced by genetic polymorphisms may have impact on the methylation status and carcinogenesis.^13^^,^^14^ The relationship between DNA methylation of P16, MGMT and hMLH1 genes and MTHFR polymorphisms with the cancer risk has been pointed in several studies.^13^^,^^15^^-^^17^ However, the evidences of DNA methylation with gastric cancer risk and relation to MTHFR are still lacking. The findings from the present study indicated that a higher hypermethylation of P16, MGMT and hMLH1 gene in gastric cancer tissues than remote-normal-appearing gastric tissues. Hypermethylation of P16 and MGMT genes was related with TNM stage in gastric cancer tissues. The DNA hypermethylation of MGMT genes present a significantly interaction with polymorphisms of MTHFR C677T.

Aberrant methylation, global hypomethylation in genomic DNA and hypermethylation in specific gene promoters, usually occurs in cancers.^18^ Lack of global DNA methylation may cause the instability of gene, and thus promote the process of cancer development.^19^ However, promoter hypermethylation may induce the inactivity of transcriptional gene.^19^ Published data indicated that the DNA methylation primarily influences the cytosine of symmetrical dinucleotide CpG in human^20^ and the subsequent pattern of DNA methylation is transmitted through mitosis and maintained after DNA replication,^21^ and thereby aberrant CpG island methylation could promote the carcinogenesis. It has been previously reported that P16, COX2, MGMT, hMSH2 and hMLH1 gene could be more frequently found in cancer tissues than in remote normal-appearing tissues, and hypermethylation could not be found in normal tissues.^13^^,^^14^^,^^22^ The present study has shown that individuals with methylation of MGMT and hMLH1 may influence the susceptibility of gastric cancer, which proved DNA methylation may play a role in the development of gastric cancer.

Our study showed that hypermethylation of P16 and MGMT genes was related with TNM stage in gastric cancer tissues. Previous study indicated that aberrant methylation of the MGMT gene was significantly correlated with the extent of tumor, lymph node metastasis, and TNM stage, and this study has shown a trend toward large maximal tumor size in methylation tumors.^23^ Our study was in line with this report. Further studies are still needed to clarify their association.

In the present study, we found polymorphisms in MTHFR C677T may influence the DNA methylation status. The main reason might be the activity of folate metabolic enzyme which participates into the methylation process of DNA. Previous studies reported that individuals carrying variant genotypes CT or TT had a higher risk of methylation of MGMT in cancer tissues.^13^^,^^22^ Only one study conducted in China indicated that MTHFR 677 T allele increased the risk of methylation of MGMT in gastric cancer tissues. Our results are in line with previous study. Taken together, these data suggest that MTHFR C677T polymorphism plays an important role in DNA methylation and carcinogensis process.

In conclusion, we found the aberrant hypermethylation of cancer-related genes, such as P16, MGMT and HMLH1, could be predictive biomarkers for detecting of gastric cancer. The aberrant hypermethylation of P16 and MGMT gene was associated with regional lymph node metastasis, and the polymorphism of MTHFR C677T could influence the methylation of MGMT. Further large-scale studies are required to elucidate the association between P16, MGMT and HMLH1 and risk of gastric cancer.
